# Digital-Use/Efficiency Phenotypes Versus Professional Role in Explaining Perceived Digital Benefit in Anaesthesia and Intensive Care

**DOI:** 10.3390/healthcare14193306

**Published:** 2026-10-03

**Authors:** Corina Vernic, Ovidiu Bedreag, Dorina Cristina Sulițanu, Liliana Ioana Muntean, Sorin Ursoniu

**Affiliations:** 1 Doctoral School, “Victor Babeș” University of Medicine and Pharmacy Timișoara, Eftimie Murgu Square 2, 300041 Timișoara, Romania; dorina.beck@umft.ro; 2 Medical Informatics and Biostatistics, Department III: Functional Sciences, Faculty of Medicine, “Victor Babeș” University of Medicine and Pharmacy Timișoara, 300041 Timișoara, Romania; 3 Research Center in Anesthesia and Intensive Care (CCATITM), Faculty of Medicine, “Victor Babeș” University of Medicine and Pharmacy Timișoara, 300041 Timișoara, Romania; bedreag.ovidiu@umft.ro; 4 Discipline of Anaesthesia and Intensive Care, Department X: Surgery II, Faculty of Medicine, “Victor Babeș” University of Medicine and Pharmacy Timișoara, 300041 Timișoara, Romania; 5 Discipline of Cellular and Molecular Biology, Department II: Microscopic Morphology, Faculty of Medicine, “Victor Babeș” University of Medicine and Pharmacy Timișoara, Eftimie Murgu Square 2, 300041 Timișoara, Romania; muntean.ioana@umft.ro; 6 Public Health and Management, Department III: Functional Sciences, Faculty of Medicine, “Victor Babeș” University of Medicine and Pharmacy Timișoara, 300041 Timișoara, Romania; sursoniu@umft.ro; 7 Centre for Translational Research and Systems Medicine, Faculty of Medicine, “Victor Babeș” University of Medicine and Pharmacy Timișoara, 300041 Timișoara, Romania

**Keywords:** latent class analysis, health personnel, attitude to computers, medical informatics, critical care

## Abstract

**Background and Objectives**: We compared professional role with digital-use/efficiency phenotype as explanations of perceived digital benefit in anaesthesia and intensive care. **Materials and Methods**: Cross-sectional survey data from 161 professionals were analysed. Latent class analysis used 12 indicators of technology/educational-resource use and perceived operational efficiency; four benefit items remained external outcomes. Although BIC favoured two classes, a three-class exploratory solution was retained using aBIC, entropy, bootstrap testing, class size and interpretability. Nested logistic models with seniority retained in every model, likelihood-ratio tests and Shapley partitioning of McFadden’s pseudo-R^2^ compared role and phenotype; pre-planned sensitivity analyses re-estimated the comparison under two- and four-class solutions, with classification-error (BCH) correction and with use-only indicators, and discrimination was internally validated by bootstrap. Descriptive decomposition and Rasch/DIF analyses were exploratory. **Results**: The three phenotypes (*n* = 83, 44 and 34; entropy of 0.815) crossed professional boundaries (Cramér’s V = 0.439). Phenotype did not improve seniority-and-role models for broad benefit (likelihood ratio 2.4, *p* = 0.301) or high uncertainty (2.8, *p* = 0.248), whereas adding role to seniority-and-phenotype models improved fit (23.6 and 26.2; both *p* < 0.001); this pattern was unchanged across all sensitivity specifications. Role received 68.3% and 62.3% of the Shapley-partitioned pseudo-R^2^, versus 11.9% and 11.4% for phenotype. Exploratory decompositions provided little evidence of an indirect association through adoption breadth or phenotype. Rasch analysis suggested patient safety was easiest and managerial benefit hardest to endorse; person separation reliability was 0.07, and DIF contrasts were descriptive. **Conclusions**: In this sample, professional role carried more explanatory information about perceived benefit than the exploratory phenotype, and this ranking was robust to class enumeration and classification-error correction. The phenotype findings remain exploratory, and cross-sectional associations do not establish causation.

## 1. Introduction

The digital transformation of anaesthesia and intensive care is now understood as a sociotechnical process rather than a technical one, in which the workforce determines whether a deployed system delivers value or is quietly abandoned [1]. Frameworks of technology acceptance have shown repeatedly that perceived usefulness and perceived ease of use, rather than the objective capabilities of a system, govern sustained use [2], and that these constructs behave differently in healthcare from the commercial settings in which they were developed [3]. Syntheses published since the pandemic confirm that these determinants remain decisive: an umbrella review of barriers and facilitators to health professionals’ use of digital technologies identified usability, workflow fit, training and organisational support, rather than the technology itself, as the recurrent determinants of sustained use [4], a conclusion echoed by recent systematic reviews of technology acceptance in healthcare [5] and by the WHO Global Strategy on Digital Health 2020–2025, which treats workforce readiness as a precondition for digital transformation [6]. When an institution evaluates its own digital maturity, it therefore measures two things at once: what its staff actually do with the available tools, and what they believe those tools accomplish. The relation between the two is usually assumed rather than examined.

Professional role is a plausible starting point for examining these differences because nurses, residents and specialists encounter digital systems through different tasks, responsibilities and decision rights. However, role may not be the only meaningful unit of analysis. Individuals within the same professional category may differ substantially in the technologies and educational resources they use and in the operational efficiencies they perceive. Grouping respondents solely by title can therefore obscure cross-cutting patterns of digital engagement that may be more closely related to benefit appraisal.

That choice is defensible but not self-evident. The diffusion-of-innovations literature describes adopter categories defined by behaviour rather than by occupational title, and predicts that individuals within the same occupational group will distribute themselves across those categories [7]. Interpretive reviews of health-information-technology implementation similarly locate the determinants of adoption in local work systems, incentives and exposure rather than in professional identity as such [8]. If that is correct, the respondents in a single department should form engagement profiles that partially cross the nurse–physician boundary, and those profiles might carry information about appraisal that professional category alone does not.

Studies of nursing staff report heterogeneity in attitudes toward electronic records and identify computer experience, perceived usefulness, training, and work context as correlates [9,10]. More recent evidence points the same way: documentation burden is measured and experienced differently by physicians and nurses [11]; nurses exposed to recent electronic health record implementations with insufficient training report more stress, time pressure and cognitive failures [12]; and reviews of nurses’ digital competence emphasise that skills, role expectations and system design jointly shape how nurses experience the same tools [13]. These studies do not by themselves establish a nurse–physician contrast, but they show why occupational groups may encounter the same technology differently. Work-system models place the person within a configuration of tasks, tools, organisation and environment [14]. Professional role may therefore act as a proxy for different digital work systems, while phenotype may capture cross-cutting patterns within those systems.

The clinical setting also matters. Anaesthesia and intensive care are among the most data-intensive hospital environments, and the range of tools available to their staff has widened rapidly since 2020 to include artificial intelligence (AI)-based applications for risk prediction, monitoring and decision support in the intensive care unit and along the perioperative pathway [15,16,17]. Surveys of intensivists and anaesthesiologists report broadly favourable but conditional attitudes toward such tools, with recurring concerns about transparency, workflow integration and accountability [18,19,20]. Whether a digital tool is perceived as beneficial therefore appears to depend on who uses it and in which task context, which is the question the present study addresses.

Evidence on digital efficiency in intensive care has so far been generated mainly at the level of systems rather than of the people who use them. A randomised comparison of paper and electronic documentation found that a clinical information system reduced nursing documentation time from 20.5% to 14.4% of total nursing time, with the recovered time redirected to direct patient care [21], and a review of critical care information systems concluded that such time savings are consistent for nurses but inconsistent for physicians [22]. Implementation of an intensive care information system has been associated with a shorter length of stay [23], and an electronic rounding checklist reduced provider workload and errors [24]. At the same time, a survey of 535 intensive care nurses and physicians found that usability differed markedly across eight commercial systems [25], and a recent qualitative study of intensive care staff catalogued 86 distinct usability problems, most of them concerning information presentation and documentation steps [26]. These studies establish that the efficiency and burden of the same tools differ by task and by user group, in keeping with the fit between individual, task and technology described by the FITT framework [27], but they do not ask whether the patterns of use and perceived efficiency reported by individual staff members, modelled as latent profiles, explain how those staff members appraise digital benefit, or whether such profiles carry information beyond professional role. The present study addresses that gap with a symmetric, pre-specified comparison of role and phenotype in a single anaesthesia and intensive care department, in a Central and Eastern European health system from which staff perspectives on digitalisation have rarely been reported. Its significance is practical: if engagement profiles explain appraisal beyond role, training and implementation support should be targeted at profiles; if role dominates, they should be organised around role-specific tasks and information needs.

These accounts lead to different associational expectations. If the digital-use/efficiency phenotype contributes information beyond professional role, the phenotype should improve models that already contain role and seniority. If professional role contributes information beyond phenotype, the reverse increment should be larger. Either pattern could reflect differences in tasks, access, exposure type or visibility of system outputs; the cross-sectional design cannot identify an immutable role effect or a causal mechanism. The practical value of the comparison is therefore to identify where explanatory information is concentrated and which mechanisms warrant prospective testing.

This study analyses respondent-level survey data from 161 anaesthesia and intensive care professionals to determine which construct provides greater explanatory information for perceived digital benefit: professional role or a latent digital-use/efficiency phenotype. Four hypotheses were specified in advance and are listed below; the analysis and the results section that address each hypothesis are given in Section 2.4.

**H1 (cross-cutting phenotypes).**  *The 12 class indicators would yield at least two substantively interpretable digital-use/efficiency phenotypes, each containing members of more than one professional group.*

**H2 (phenotype beyond role).**  *Phenotype would improve models of benefit appraisal that already contain seniority and professional role.*

**H3 (role beyond phenotype).**  *Professional role would improve models of benefit appraisal that already contain seniority and phenotype.*

**H4 (indirect association).**  *Technology-adoption breadth or the posterior probability of class 1 (comprehensive-adopter) membership would show a statistical indirect association linking physician status to appraisal.*

The latent class enumeration (H1) and the symmetric role-versus-phenotype model comparisons (H2 and H3) were primary; Shapley partitioning was secondary; the descriptive decomposition (H4) and the Rasch/DIF analyses were exploratory.

## 2. Materials and Methods

### 2.1. Study Design, Participants and Original Dataset

This single-centre cross-sectional study analysed original survey data collected from anaesthesia and intensive care personnel in a tertiary-level emergency hospital during a period of active digital infrastructure expansion. Data were collected between 4 April and 30 June 2022 in a single wave, with one administration per person; no earlier survey had been conducted in the department, so all respondents were first-time participants. All 214 personnel working in the department during the survey window (73 nurses, 78 residents and 63 specialist physicians) were invited, and participation was voluntary and anonymous. Of the 176 questionnaires returned (82.2% of those invited), 15 were excluded before analysis because at least one of the 12 class-defining indicators or one of the four benefit-appraisal items had been left blank (nine nurses, four residents and two specialists); no questionnaire was excluded for any other reason, and no imputation was performed. The analytic dataset therefore contains 161 complete questionnaires (75.2% of those invited) from 55 nurses (75.3% of invited nurses), 62 residents (79.5%) and 44 specialist physicians (69.8%). Among these 161 records, the only item-level missingness was in the daily-tool-use item (three respondents), which was handled by complete-case analysis in the analyses that used it. The full questionnaire is provided in Appendix A. For analyses contrasting nurses with physicians, residents and specialists were combined as physicians (*n* = 106). All reported analyses used these respondent-level records.

The analytic dataset retained each participant’s responses for professional role, seniority, daily digital-tool use, the four benefit-appraisal items, use of individual technologies and educational resources, the three barrier items and the five resource-efficiency items. Seniority was coded in four ordered bands: 0–5, 6–10, 11–20, and more than 20 years. Because residents were concentrated in lower seniority bands, role and seniority were treated as related adjustment variables; their coefficients were interpreted as conditional associations rather than independent causal effects. Composite indices and subsequent analyses were calculated from the respondent-level observations (Table 1).

### 2.2. Survey Instrument, Indicators, Composite Measures and the Separation of Predictors from Outcomes

The questionnaire (Appendix A) was developed by the research team in 2021 for a departmental assessment of digital maturity, because no validated instrument covered the specific inventory of systems and educational resources available in the department. Item content was derived from the perceived-usefulness and facilitating-conditions constructs of technology-acceptance theory [2,3], from the workforce domain of the WHO digital health strategy [6] and from the departmental inventory of deployed systems. Content validity was assessed by a panel of five experts (two intensivists, one anaesthesia nurse, one medical informatician and one hospital manager), who rated the relevance of each item on a four-point scale; all retained items had an item-level content validity index of at least 0.80, and the scale-level index (averaging method) was 0.93 [28]. The instrument was piloted with 12 staff members who were not part of the analytic sample, leading to minor wording changes and to the addition of the ‘cannot appraise’ option for the four benefit items. Because the seven use items and the five perceived-efficiency items are binary checklists rather than reflective scales, internal consistency is reported descriptively only: the Kuder–Richardson KR-20 coefficient was 0.72 for the seven use items and 0.74 for the five efficiency items [29]. Latent class analysis does not require unidimensional scales; it models the joint distribution of the indicators under conditional independence within classes, and model adequacy was judged from the enumeration statistics in Section 3.2 (Table 2). Test–retest reliability was not assessed and is noted as a limitation.

Twelve binary indicators were used to define the latent classes. Seven described reported use: four core technologies (patient-monitoring systems, electronic health records, anaesthesia-planning software and training simulators) and three digital educational resources (e-learning platforms, simulation-based training and webinars). Five described resources were perceived to be used more efficiently after digitalisation (medications, medical staff, sanitary materials, bed occupancy and protective equipment). Because these indicators combine behaviour with perceived operational efficiency, the latent construct is termed a latent digital-use/efficiency class (hereafter, digital-use/efficiency phenotype). The word phenotype is used descriptively for a response profile, not as a biological or stable personal trait. The five efficiency indicators also create conceptual overlap with appraisal and with the Resource-Efficiency Index; excluding the four benefit outcomes prevents direct circularity but does not make the phenotype a pure measure of exposure. To separate use-based from appraisal-based constructs as far as the design allows, a second latent class model restricted to the seven reported-use indicators was fitted as a sensitivity analysis (Section 2.3). Terminology is used consistently hereafter: ‘reported use’ denotes the seven use indicators, ‘perceived efficiency’ denotes the five resource items, ‘benefit appraisal’ denotes the four external outcome items, and the three phenotypes are referred to by class number, with the descriptive labels given in Table 3.

The four trichotomous benefit items assessed whether digital technologies improved patient safety (item Q5 of the original instrument), clinical decision-making (Q9), managerial decision-making (Q10), and continuing education (Q11). These items were deliberately excluded from the class-defining model. This separation is the central design feature of the analysis: if the appraisal items were allowed to inform class membership, any subsequent association between class and appraisal would be circular. Retaining them outside the model leaves them available as external outcomes. From the original item-level responses, we calculated the Digital Benefit Index, which counts affirmative responses across the four domains, and the Uncertainty Index, which counts ‘cannot appraise’ responses across the same four domains; each ranges from 0 to 4. The technology-adoption score (0–4), Barrier Burden score (0–3), and Resource-Efficiency Index (0–5) were also calculated directly from the corresponding questionnaire items. Broad perceived benefit was defined as a Digital Benefit Index of at least 3, and high uncertainty as an Uncertainty Index of at least 2, for the analyses reported here.

### 2.3. Latent Class Analysis and Class Enumeration

Latent class models with one to five classes were fitted to the 12 binary indicators by maximum likelihood using the expectation-maximisation algorithm with 60 random starts per solution; convergence was declared when the change in log-likelihood fell below 10^−9^. Enumeration considered AIC, BIC, sample-size-adjusted BIC (aBIC) [30], normalised entropy, smallest-class size and a parametric bootstrap likelihood-ratio test with 150 replicates [31], following published guidance on latent class analysis in critical care research [32]. BIC favoured two classes, whereas aBIC and entropy continued to improve through four classes. The three-class solution was retained as an exploratory, parsimonious compromise because it added a substantively distinct profile while avoiding the 14-person smallest class in the four-class model. This decision does not identify a uniquely true number of classes. Because the enumeration criteria disagreed, the primary role-versus-phenotype models were re-estimated under the two- and four-class solutions as pre-planned sensitivity analyses (Section 2.4), so that the main comparison could be evaluated across the plausible range of class solutions rather than conditioned on a single choice.

Respondents were assigned to their modal class for the descriptive analyses, and mean posterior assignment probabilities are reported by class. Because modal assignment ignores residual classification uncertainty and can attenuate or distort associations with external outcomes [33], the primary outcome models were additionally estimated with the bias-adjusted three-step (BCH) approach, in which respondents enter the outcome model with weights derived from the inverse of the estimated classification-error matrix [34,35], and with posterior-probability weighting, in which each respondent contributes to every class in proportion to their posterior membership probability. Standard errors for the BCH models were obtained from 500 bootstrap resamples of the complete two-stage procedure. In a further sensitivity analysis, the latent class model was re-fitted using only the seven reported-use indicators, so that the class-defining information contained no perceived-efficiency content that could overlap conceptually with benefit appraisal; the three-class use-only solution was carried into the same outcome models. Descriptive class comparisons that use modal assignment remain exploratory. Associations between class and professional role, seniority band and daily tool use were tested with Pearson chi-square tests and quantified with Cramér’s V, a normalised chi-square coefficient of association that does not represent a proportion of explained variance [36]. Adjusted contrasts of classes 2 and 3 against class 1 used Firth-penalised binary logistic regression [37] because sparse or empty cells prevented stable ordinary maximum-likelihood estimation.

### 2.4. Competing Explanations, Item Response Modelling and Statistical Analysis

**Figure 1:**
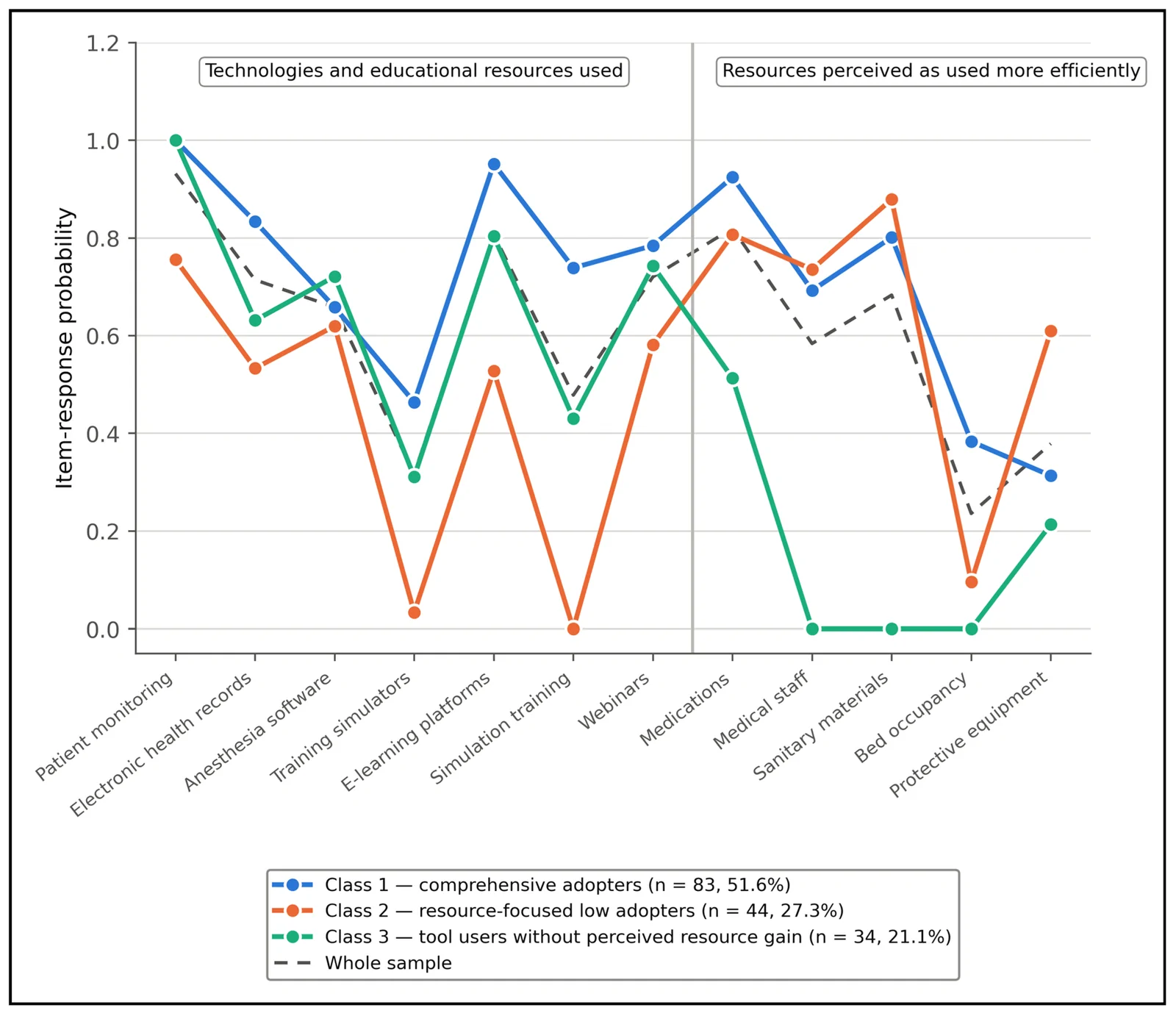
Item-response probabilities for the selected three digital-use/efficiency phenotypes across the 12 class-defining indicators, with whole-sample prevalence shown for reference.

**Figure 2:**
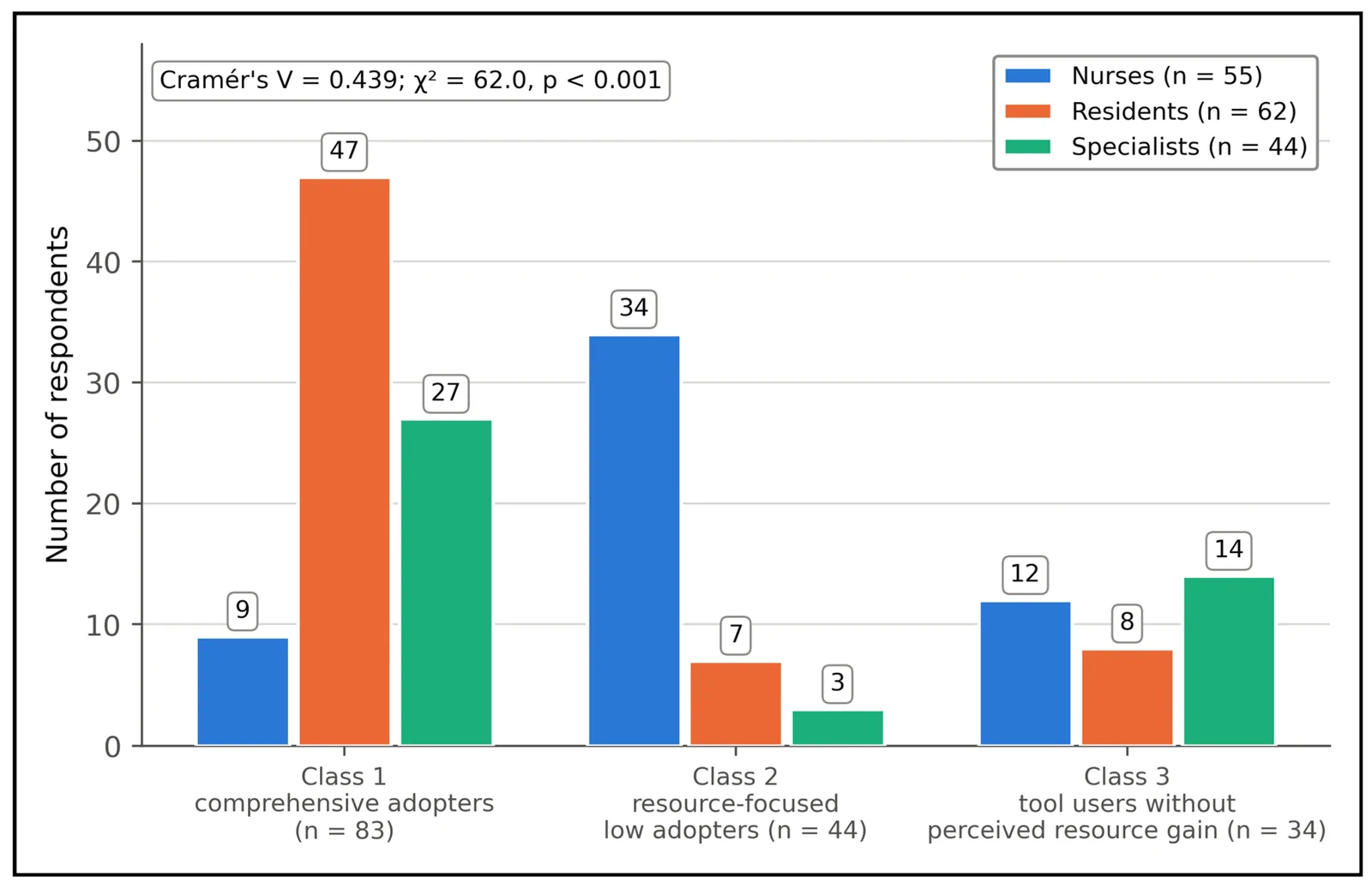
Composition of each digital-use/efficiency phenotype by professional role, with respondent counts shown above each bar.

Each hypothesis was linked in advance to a specific analysis, decision rule and reporting section, as follows:

**H1.** Latent class enumeration (Section 2.3; Table 2), the item-response profiles (Table 3, Figure 1) and the cross-classification of modal class with professional role using the Pearson chi-square test, Cramér’s V and Firth-penalised contrasts (Table 4, Figure 2). H1 was considered supported if at least two interpretable classes were retained and every class contained members of more than one profession (Section 3.2, Section 3.3 and Section 3.4). The external associations of the phenotypes with benefit appraisal, barriers and the composite measures (Tables 5 and 6, Section 3.5 and Section 3.6) were descriptive and were not hypothesis tests.

**H2.** The likelihood-ratio test for adding the two phenotype indicators to the seniority-and-role model (M3 versus M1, defined below) for each primary outcome, with Holm adjustment across the four primary tests; support required *p* < 0.05 after adjustment (Tables 7 and 8, Section 3.7). The result was re-examined under the sensitivity specifications of Section 2.3 (Table 9, Section 3.8).

**H3.** The likelihood-ratio test for adding the two role indicators to the seniority-and-phenotype model (M3 versus M2) under the same decision rule (Tables 7 and 9, Section 3.7 and Section 3.8). Shapley partitioning of McFadden’s pseudo-R^2^ (Section 3.10) was a secondary descriptive comparison of the two constructs, and the exploratory Rasch/DIF analysis of the four benefit items (Table 10, Section 3.9) was not linked to a pre-specified hypothesis.

**H4.** The exploratory descriptive decomposition of the nurse–physician contrast, with technology-adoption score and class 1 posterior probability as candidate intermediate variables (Table 11, Section 3.10); an indirect component whose bootstrap confidence interval excluded zero would have been taken as exploratory support.

Appraisal outcomes were compared across classes with the Kruskal–Wallis test for ordinal indices, epsilon-squared effect sizes and Bonferroni-corrected Mann–Whitney U tests for pairwise contrasts, and with Pearson chi-square or Fisher exact tests for binary items. The primary explanatory comparison used four nested logistic models for broad perceived benefit and high uncertainty, with seniority band retained in every model so that the two constructs were compared symmetrically: a seniority-only base model (M0), a seniority-and-role model (M1), a seniority-and-phenotype model (M2) and a combined model (M3). The incremental contribution of phenotype was assessed by comparing M2 with M0 and M3 with M1, and that of role by comparing M1 with M0 and M3 with M2; each increment therefore isolates a single construct with 2 degrees of freedom. Item-specific safety and educational-benefit models were secondary. Likelihood-ratio tests were the primary basis for assessing incremental contribution, supplemented by AIC and BIC. Because the DeLong test is invalid for comparing the C-statistics of nested models fitted and evaluated in the same sample [38], DeLong *p*-values are not reported; differences in the C-statistic are instead accompanied by 95% confidence intervals from 1000 bootstrap resamples in which both models were re-fitted, and each model’s apparent C-statistic is accompanied by an optimism-corrected value obtained with Harrell’s bootstrap procedure using 200 resamples [39]. Adjusted odds ratios with Wald 95% confidence intervals are reported for the seniority-and-role and combined models. Hierarchical partitioning allocated the total McFadden pseudo-R^2^ among role, phenotype, technology-adoption score and seniority as Shapley values averaged over all entry orders [40]. These values are reported as shares of Shapley-partitioned McFadden pseudo-R^2^, not as proportions of outcome variance. Because phenotype, adoption score and efficiency indicators overlap conceptually and empirically, the allocations describe correlated predictor blocks rather than independent mechanisms.

An exploratory descriptive decomposition partitioned the model-based nurse–physician contrast, with residents and specialists combined as physicians, into a direct component and an indirect component transmitted through the candidate intermediate variable, on the risk-difference scale. The technology-adoption score and the posterior probability of class 1 (comprehensive-adopter) membership were evaluated separately, adjusting for seniority band, with 800 bootstrap replications and 400 intermediate-variable simulations per replication [41]. The computational algorithm follows the counterfactual mediation literature [41], but the quantities are reported as descriptive components of an observed association rather than as natural direct and indirect effects or proportions mediated, because causal interpretation would require temporal ordering and no unmeasured exposure–intermediate, intermediate–outcome or exposure–outcome confounding, none of which can be established in a cross-sectional survey. The percentage of the total contrast carried by the indirect component is reported only as a descriptive ratio. With 800 bootstrap replications, the smallest attainable two-sided percentile *p*-value is 0.0025, and smaller values are reported as *p* < 0.003. The four benefit items were then examined exploratorily with Rasch and two-parameter item-response models [42]. DIF values were obtained from group-centred nurse and physician item difficulties. Because standard errors, confidence intervals, *p*-values and multiplicity-adjusted tests were not available for the DIF contrasts, they are descriptive signals only. Exact two-sided *p*-values and 95% confidence intervals are reported where available. Multiplicity was handled at two levels. The four pre-specified primary likelihood-ratio tests (the phenotype and role increments for each of the two primary outcomes) form a single family and are reported with Holm-adjusted *p*-values [43]. Secondary and exploratory tests (the item-level and barrier comparisons in Table 5, the secondary item-specific model increments and the exploratory decomposition components) are reported with Benjamini–Hochberg false-discovery-rate-adjusted q-values within their family [44], in addition to the Bonferroni-corrected pairwise contrasts; exploratory findings remain hypothesis-generating regardless of adjustment. Analyses were performed in Python 3.11 with NumPy, SciPy, pandas and statsmodels; the BCH, bootstrap and Shapley procedures were implemented in the same environment, and the scripts are available as described in the Data Availability Statement.

## 3. Results

The results are organised around the four hypotheses. Section 3.1 provides descriptive context from the composite indices and their correlations. Section 3.2, Section 3.3 and Section 3.4 address H1 (class enumeration, description of the phenotypes and their cross-classification with professional role). Section 3.5 and Section 3.6 describe the external associations of the phenotypes and are not hypothesis tests. Section 3.7 addresses H2 and H3 in the primary nested models, and Section 3.8 reports the sensitivity analyses for the same comparison. Section 3.9 reports the exploratory Rasch analysis, and Section 3.10 addresses H4 and the secondary Shapley partitioning.

### 3.1. Composite Measures and Correlations in the Original Dataset

Composite-index values by professional group and selected associations calculated directly from the original respondent-level records are summarised in Table 1.

**Table 1:** Composite-index values and selected correlations calculated from the respondent-level dataset (*N* = 161).

Measure or Association	Population	Observed Estimate	Statistic
**Composite index means by professional group**			
Digital Benefit Index	Nurses	2.31	Mean
Digital Benefit Index	Residents	3.53	Mean
Digital Benefit Index	Specialists	3.36	Mean
Technology-adoption score	Nurses	2.02	Mean
Technology-adoption score	Residents	3.03	Mean
Technology-adoption score	Specialists	2.80	Mean
Barrier Burden score	Nurses	1.87	Mean
Barrier Burden score	Residents	1.94	Mean
Barrier Burden score	Specialists	1.86	Mean
Resource-Efficiency Index	Nurses	2.60	Mean
Resource-Efficiency Index	Residents	3.05	Mean
Resource-Efficiency Index	Specialists	2.34	Mean
Uncertainty Index	Nurses	1.60	Mean
Uncertainty Index	Physicians	0.49	Mean
**Associations in the full sample**			
Digital Benefit Index vs. technology-adoption score	All respondents	+0.25	Spearman rho
Digital Benefit Index vs. seniority band	All respondents	−0.17	Spearman rho
Technology-adoption score vs. seniority band	All respondents	−0.20	Spearman rho
Clinical vs. managerial benefit	All respondents	+0.31	Spearman rho
Safety vs. educational benefit	All respondents	+0.29	Spearman rho
**Association within nurses**			
Seniority band vs. Uncertainty Index	Nurses	+0.42	Spearman rho

Values are presented as means for composite indices and as Spearman rho coefficients for associations. All values were calculated directly from the study’s original respondent-level survey data. The within-nurse association uses the nursing subgroup. Abbreviations: none.

Mean Digital Benefit Index values were 2.31 for nurses, 3.53 for residents, and 3.36 for specialists. The corresponding technology-adoption means were 2.02, 3.03 and 2.80; Barrier Burden means were 1.87, 1.94 and 1.86; and Resource-Efficiency Index means were 2.60, 3.05 and 2.34. The mean Uncertainty Index was 1.60 among nurses and 0.49 among physicians. In the full sample, the Digital Benefit Index correlated with technology adoption (Spearman rho = 0.25) and seniority (rho = −0.17), while technology adoption correlated with seniority (rho = −0.20). Correlations between clinical and managerial benefit (rho = 0.31) and between safety and educational benefit (rho = 0.29) were modest. Within nurses, seniority correlated with uncertainty (rho = 0.42).

### 3.2. Class Enumeration: How Many Digital-Use/Efficiency Phenotypes? (H1)

Latent class models with one to five classes were fitted to the 12 digital-use/efficiency indicators, and the enumeration statistics are shown in Table 2.

**Table 2:** Latent class enumeration for one- to five-class models fitted to the 12 digital-use/efficiency indicators (*N* = 161).

k	Log L	No. Par.	AIC	BIC	aBIC	Entropy	Smallest Class, *n* (%)	Bootstrap LRT vs. k − 1
1	−1106.7	12	2237.3	2274.3	2236.3	1.000	161 (100.0)	—
2	−1062.6	25	2175.2	2252.3	2173.1	0.699	60 (37.3)	88.1; *p* = 0.007
3	−1030.8	38	2137.6	2254.7	2134.4	0.815	34 (21.1)	63.7; *p* = 0.007
4	−1014.7	51	2131.4	2288.5	2127.1	0.902	14 (8.7)	32.2; *p* = 0.047
5	−1002.3	64	2132.6	2329.8	2127.2	0.934	15 (9.3)	—

Models were fitted by expectation-maximisation with 60 random starts. Lower AIC, BIC and aBIC indicate better fit; entropy is normalised. The bootstrap likelihood-ratio test used 150 parametric replicates, so the smallest attainable *p*-value is 0.007. BIC favoured two classes, while aBIC and entropy continued to improve through four classes. The three-class solution was retained as an exploratory, parsimonious working model based on its distinct third profile, entropy, bootstrap test and class sizes; it is not presented as the uniquely supported solution. The bootstrap test was not run for the five-class comparison because adjusted criteria had ceased to improve at four classes. Abbreviations: aBIC, sample-size-adjusted Bayesian information criterion; AIC, Akaike information criterion; BIC, Bayesian information criterion; k, number of classes; Log L, log-likelihood; LRT, likelihood-ratio test; No. par., number of estimated parameters.

The enumeration criteria disagreed. BIC reached its minimum at two classes (2252.3), whereas AIC and aBIC continued to fall through four classes and the bootstrap likelihood-ratio test remained significant for the four- versus three-class comparison (32.2; *p* = 0.047). Entropy increased from 0.699 for two classes to 0.815 for three and 0.902 for four. The four-class solution divided the largest class into two profiles differing mainly in degree and produced a 14-person smallest class; the five-class solution did not improve aBIC (2127.2 versus 2127.1). The three-class solution was therefore used as an interpretable compromise. It identified a third profile not represented in the two-class solution. Because the choice remains uncertain, the primary outcome models were re-estimated under the two- and four-class solutions (Section 3.8).

### 3.3. Description of the Digital-Use/Efficiency Phenotypes (H1)

The item-response probabilities describing the selected three-class solution are given in Table 3 and displayed against the sample prevalence of each indicator in Figure 1.

**Table 3:** Item-response probabilities for the selected three-class digital-use/efficiency solution, with observed indicator prevalence.

Indicator	Whole Sample, %	Class 1 (*n* = 83)	Class 2 (*n* = 44)	Class 3 (*n* = 34)
**Technologies and educational resources used**				
Patient-monitoring systems	93.2	1.00	0.76	1.00
Electronic health records	71.4	0.83	0.53	0.63
Anaesthesia-planning software	65.8	0.66	0.62	0.72
Training simulators	31.7	0.46	0.03	0.31
E-learning platforms	80.7	0.95	0.53	0.80
Simulation-based training	47.8	0.74	0.00	0.43
Webinars	72.0	0.78	0.58	0.74
**Resources perceived as used more efficiently**				
Medications	82.0	0.92	0.81	0.51
Medical staff	58.4	0.69	0.74	0.00
Sanitary materials	68.3	0.80	0.88	0.00
Bed occupancy	23.6	0.38	0.10	0.00
Protective equipment	37.9	0.31	0.61	0.21

Values are model-estimated conditional probabilities of endorsing each indicator given class membership. Class 1 is labelled comprehensive adopters, class 2 is labelled resource-focused low adopters, and class 3 is labelled tool users without perceived resource gain. These labels summarise observed response profiles and do not imply stable personal traits or causal effects. Class prevalences were 51.6%, 27.3% and 21.1%; mean posterior assignment probabilities were 0.95, 0.91 and 0.83, respectively, and normalised entropy was 0.815. The four benefit-appraisal items were excluded from the model. Abbreviations: none.

Class 1 included 83 respondents (51.6%) and showed high probabilities for most use indicators, including monitoring systems (1.00), electronic records (0.83), e-learning (0.95) and simulation-based training (0.74), together with comparatively high perceived efficiency gains for medications and bed occupancy. Class 2 included 44 respondents (27.3%) and combined low reported use of training simulators (0.03) and simulation-based training (0.00) with high perceived gains for selected resources, particularly sanitary materials (0.88) and protective equipment (0.61). This pattern is descriptive and does not establish that low simulation use produced those perceived gains. Class 3 included 34 respondents (21.1%) and showed intermediate-to-high tool use but probabilities of 0.00 for perceived efficiency gains in medical staff, sanitary materials, and bed occupancy. Mean posterior assignment probabilities were 0.95, 0.91, and 0.83; the lower value for class 3 and the use of modal assignment warrant caution in downstream comparisons.

Across the seven use indicators, class 1 probabilities were generally above the sample average and class 2 probabilities were generally below it, with the largest separations at the two simulation-related indicators. Class 3 was closer to class 1 on several use indicators. Across the five perceived-efficiency indicators, class 3 had probabilities of 0.00 for medical staff, sanitary materials, and bed occupancy, while classes 1 and 2 had higher probabilities for medical staff and sanitary materials. Thus, the third profile was distinguished mainly by the combination of tool use and low reported operational-efficiency gains.

### 3.4. Association Between Digital-Use/Efficiency Phenotype and Professional Role (H1)

The distribution of the digital-use/efficiency phenotypes across professional categories, seniority bands and daily tool use is reported in Table 4 and shown in Figure 2.

**Table 4:** Distribution of the three digital-use/efficiency phenotypes across professional role, seniority, and daily tool use, with adjusted contrasts.

Characteristic	Class 1 (*n* = 83)	Class 2 (*n* = 44)	Class 3 (*n* = 34)	Test Statistic; *p*-Value
**Panel A. Composition, *n* (row %)**				
Nurses (*n* = 55)	9 (16.4)	34 (61.8)	12 (21.8)	χ^2^ = 62.0; *p* < 0.001
Residents (*n* = 62)	47 (75.8)	7 (11.3)	8 (12.9)	Cramér’s V = 0.439
Specialists (*n* = 44)	27 (61.4)	3 (6.8)	14 (31.8)	
Seniority 0–5 years (*n* = 96)	59 (61.5)	21 (21.9)	16 (16.7)	χ^2^ = 11.9; *p* = 0.063
Seniority 6–10 years (*n* = 11)	3 (27.3)	5 (45.5)	3 (27.3)	Cramér’s V = 0.193
Seniority 11–20 years (*n* = 18)	5 (27.8)	8 (44.4)	5 (27.8)	
Seniority > 20 years (*n* = 36)	16 (44.4)	10 (27.8)	10 (27.8)	
Daily digital tool use	83 (100.0)	41 (93.2)	34 (100.0)	*p* = 0.028 †
**Panel B. Firth-penalised logistic contrasts, adjusted odds ratio (95% CI)**				
Resident vs. nurse	1 (reference)	0.03 (0.01–0.12)	0.11 (0.03–0.46)	*p* < 0.001; *p* = 0.002
Specialist vs. nurse	1 (reference)	0.04 (0.01–0.15)	0.39 (0.13–1.17)	*p* < 0.001; *p* = 0.093
Seniority band, per level	1 (reference)	0.85 (0.53–1.36)	0.92 (0.61–1.37)	*p* = 0.495; *p* = 0.674
Daily digital tool use	1 (reference)	0.05 (0.00–3.42)	not estimable ‡	*p* = 0.162; —

Panel A percentages are row percentages within each characteristic, except the daily-use row, where they are within-class percentages. † Freeman–Halton exact test; three of the 161 respondents did not report daily use, including all of them in class 2. Panel B reports separate binary logistic models contrasting class 2 with class 1 (*n* = 127) and class 3 with class 1 (*n* = 117), each with Firth penalisation because several cells are sparse or empty—only three specialists occupy class 2, and daily tool use is universal in classes 1 and 3; ‡ daily tool use has no variation in the class 3 versus class 1 comparison and is therefore not estimable there; class 1 is the reference category in both, and the final column gives the *p*-value for the class 2 and class 3 contrasts respectively. Model C-statistics were 0.840 and 0.705. Abbreviations: CI, confidence interval.

Phenotype and professional role were strongly associated but far from identical. The chi-square statistic was 62.0 on four degrees of freedom and Cramér’s V was 0.439, a moderate-to-strong association on conventional benchmarks [36]. Because Cramér’s V is a normalised chi-square coefficient rather than a proportion of explained variance, it should not be read as the share of class membership accounted for by role; the cross-classification itself shows why the two are not interchangeable. Nine nurses, 16.4% of the nursing group, fell in class 1 (comprehensive adopters) alongside the physician majority. Thirty-two of the 106 physicians (30.2%) fell outside the class that dominated their profession: ten in class 2 (resource-focused low adopters; seven residents and three specialists) and twenty-two in class 3 (tool users without perceived resource gain; eight residents and fourteen specialists). Class 3 was the most clearly cross-cutting, drawing 12 nurses, 8 residents and 14 specialists, so that it contained members of all three professions in appreciable numbers rather than being dominated by one. In the adjusted contrasts, professional category remained the dominant correlate of membership, with residents and specialists 25- to 33-fold less likely than nurses to occupy class 2, but seniority band was unrelated to phenotype in every contrast (odds ratios 0.85 and 0.92; *p* = 0.495 and 0.674), as was the unadjusted distribution across the four bands (Cramér’s V = 0.193; *p* = 0.063). In contrast, seniority correlated with uncertainty within nurses (Spearman rho = 0.42; Table 1) while remaining unrelated to phenotype membership.

The counts in Figure 2 show substantial but incomplete alignment between class and professional role. Class 1 contained 9 nurses, 47 residents and 27 specialists; class 2 contained 34 nurses, 7 residents and 3 specialists; and class 3 contained 12 nurses, 8 residents and 14 specialists. Class 3 therefore included all three professional categories and represented 21.8% of nurses, 12.9% of residents and 31.8% of specialists. These counts support cross-boundary membership while also showing that class composition remained strongly associated with role.

### 3.5. Benefit Appraisal and Barriers Across Digital-Use/Efficiency Phenotypes (Descriptive External Associations)

Because the four appraisal items were held outside the class-defining model, they can be examined as external outcomes, as reported in Table 5.

**Table 5:** Benefit appraisal and reported barriers by digital-use/efficiency phenotype. None of these items entered the class-defining model.

Outcome	Class 1 (*n* = 83)	Class 2 (*n* = 44)	Class 3 (*n* = 34)	Test	*p*-Value	ε^2^
Digital Benefit Index (0–4)	3.29 ± 0.96	2.91 ± 1.07	2.74 ± 1.40	H = 6.0	0.051	0.025
Uncertainty Index (0–4)	0.67 ± 0.94	1.00 ± 1.08	1.18 ± 1.38	H = 4.4	0.108	0.015
Perceived safety benefit, *n* (%)	77 (92.8)	34 (77.3)	27 (79.4)	Exact	0.027	—
Perceived clinical benefit, *n* (%)	65 (78.3)	32 (72.7)	22 (64.7)	χ^2^ = 2.36	0.307	—
Perceived managerial benefit, *n* (%)	56 (67.5)	31 (70.5)	21 (61.8)	χ^2^ = 0.67	0.716	—
Perceived educational benefit, *n* (%)	75 (90.4)	31 (70.5)	23 (67.6)	χ^2^ = 11.37	0.003	—
Barrier: lack of training, *n* (%)	40 (48.2)	23 (52.3)	19 (55.9)	χ^2^ = 0.61	0.736	—
Barrier: technical problems, *n* (%)	69 (83.1)	40 (90.9)	28 (82.4)	χ^2^ = 1.63	0.444	—
Barrier: resistance to change, *n* (%)	42 (50.6)	24 (54.5)	20 (58.8)	χ^2^ = 0.69	0.710	—
Barrier Burden score (0–3)	1.82 ± 0.83	1.98 ± 0.73	1.97 ± 0.72	H = 1.1	0.580	−0.006

Values are mean ± SD or *n* (%). Ordinal indices were compared with the Kruskal–Wallis test, whose statistic is denoted H, and binary items with the Pearson chi-square test or the Freeman–Halton exact test where any expected cell count was below five; this applied only to the perceived-safety row (minimum expected count 4.86). Bonferroni-corrected pairwise Mann–Whitney comparisons were non-significant throughout (smallest corrected *p* = 0.111, class 1 versus class 2 for the Digital Benefit Index). After Benjamini–Hochberg adjustment across the ten tests in this Table, only the educational-benefit contrast remained significant (q = 0.030); the safety contrast (q = 0.135) and the Digital Benefit Index gradient (q = 0.170) did not. Abbreviations: ε^2^, epsilon-squared effect size; H, Kruskal–Wallis statistic; SD, standard deviation.

The phenotypes differed far less in what respondents believed than in what they did. The Digital Benefit Index declined across the three classes from 3.29 to 2.91 to 2.74, a gradient that approached conventional significance (H = 6.0; *p* = 0.051) and corresponded to an epsilon-squared of 0.025. The Uncertainty Index rose from 0.67 to 1.18 without reaching significance (*p* = 0.108), and no Bonferroni-corrected pairwise contrast was significant for either index. At the item level, perceived educational benefit fell from 90.4% in comprehensive adopters to 70.5% and 67.6% in the other two classes (*p* = 0.003), and perceived safety benefit fell from 92.8% to 77.3% and 79.4% (*p* = 0.027). After false-discovery-rate adjustment across the ten comparisons in Table 5, only the educational-benefit difference remained significant (q = 0.030; safety q = 0.135). Clinical and managerial benefit did not differ across phenotypes (*p* = 0.307 and 0.716). Reported barriers were also similar across phenotypes: *p* = 0.736 for lack of training, *p* = 0.444 for technical problems, and *p* = 0.710 for resistance to change; the Barrier Burden score yielded *p* = 0.580.

### 3.6. Correlation Structure of the Composite Measures (Descriptive)

The bivariate relationships among the composite measures, seniority, and class posterior probabilities are given in Table 6.

**Table 6:** Spearman rank-correlation matrix of composite indices, seniority and class posterior probabilities (*N* = 161).

Variable	Digital Benefit Index	Uncertainty Index	Technology-Adoption Score	Resource-Efficiency Index
Digital Benefit Index	—	−0.97 (−0.98 to −0.96) *	0.25 (0.10 to 0.39) *	0.20 (0.05 to 0.34) *
Uncertainty Index	−0.97 (−0.98 to −0.96) *	—	−0.24 (−0.38 to −0.09) *	−0.19 (−0.34 to −0.04) *
Technology-adoption score	0.25 (0.10 to 0.39) *	−0.24 (−0.38 to −0.09) *	—	0.05 (−0.11 to 0.20)
Resource-Efficiency Index	0.20 (0.05 to 0.34) *	−0.19 (−0.34 to −0.04) *	0.05 (−0.11 to 0.20)	—
Barrier Burden score	0.09 (−0.07 to 0.24)	−0.10 (−0.25 to 0.05)	−0.06 (−0.21 to 0.10)	0.00 (−0.16 to 0.15)
Seniority band	−0.17 (−0.32 to −0.02) *	0.19 (0.04 to 0.34) *	−0.20 (−0.35 to −0.05) *	−0.19 (−0.33 to −0.03) *
Posterior probability of class 1	0.23 (0.08 to 0.37) *	−0.20 (−0.34 to −0.04) *	0.49 (0.36 to 0.60) *	0.36 (0.22 to 0.49) *
Posterior probability of class 2	−0.17 (−0.31 to −0.01) *	0.13 (−0.03 to 0.28)	−0.51 (−0.62 to −0.39) *	0.13 (−0.03 to 0.27)
Posterior probability of class 3	−0.17 (−0.32 to −0.02) *	0.17 (0.01 to 0.31) *	−0.06 (−0.21 to 0.09)	−0.91 (−0.94 to −0.88) *

Values are Spearman rho with 95% confidence intervals from the Fisher z-transformation, computed from the unrounded coefficient, so identical tabulated values may carry limits differing in the second decimal place. * *p* < 0.05. The near-perfect inverse correlation between the Digital Benefit Index and the Uncertainty Index is structural rather than empirical: both are counts over the same four items, and explicit disagreement was recorded only 10 times in 644 responses, so the two indices are almost complementary by construction. The correlation of −0.91 between class 3 membership and the Resource-Efficiency Index likewise reflects that the resource items help define that class. Abbreviations: none.

Several features of the matrix require qualification. The correlation of −0.97 between the Digital Benefit Index and the Uncertainty Index is structural: the two are counts of affirmative and non-appraising responses over the same four items, and explicit disagreement was recorded only ten times in 644 responses, so the indices are nearly complementary by construction. The same caution applies to the −0.91 correlation between class 3 membership and the Resource-Efficiency Index, because the resource items help define that class. Setting these aside, the informative correlations were modest. The technology-adoption score correlated 0.25 with perceived benefit and −0.24 with uncertainty, and the Resource-Efficiency Index correlated 0.20 and −0.19 with the same outcomes. Seniority correlated −0.17 with benefit and 0.19 with uncertainty. The posterior probability of class 1 (comprehensive-adopter) membership correlated 0.49 with the adoption score and 0.36 with the Resource-Efficiency Index, but only 0.23 with perceived benefit and −0.20 with uncertainty. Barrier Burden showed no meaningful correlation with the other measures (absolute rho ≤ 0.10).

### 3.7. Professional Role Versus Digital-Use/Efficiency Phenotype (H2 and H3)

The primary comparison placed professional role and digital-use/efficiency phenotype in symmetric nested logistic models with seniority retained in every model, reported in Table 7; the corresponding adjusted odds ratios are given in Table 8.

**Table 7:** Professional role and digital-use/efficiency phenotype as explanations of benefit appraisal: nested logistic models with seniority retained in every model.

Outcome and Model	Log-Likelihood	AIC/BIC	C-Statistic, Apparent (95% CI)	C-Statistic, Optimism-Corrected	Increment: LR (df; *p*); ΔAUC (Bootstrap 95% CI)
**Broad perceived benefit (DBI ≥ 3)**					
M0—seniority only	−90.9	185.8/192.0	0.586 (0.492–0.680)	0.571	reference
M1—seniority and role	−78.4	164.9/177.2	0.760 (0.674–0.846)	0.741	Role vs. M0: LR = 25.0 (2; <0.001); ΔAUC +0.174 (0.078 to 0.271)
M2—seniority and phenotype	−89.0	186.0/198.3	0.632 (0.537–0.727)	0.601	Phenotype vs. M0: LR = 3.8 (2; 0.150); ΔAUC +0.046 (−0.021 to 0.118)
M3—seniority, role and phenotype	−77.2	166.5/185.0	0.770 (0.678–0.862)	0.739	Phenotype vs. M1: LR = 2.4 (2; 0.301); ΔAUC +0.010 (−0.019 to 0.044). Role vs. M2: LR = 23.6 (2; <0.001); ΔAUC +0.138 (0.052 to 0.229)
**High diagnostic uncertainty (UI ≥ 2)**					
M0—seniority only	−85.4	174.8/181.0	0.598 (0.497–0.699)	0.579	reference
M1—seniority and role	−71.9	151.8/164.1	0.757 (0.658–0.856)	0.735	Role vs. M0: LR = 27.0 (2; <0.001); ΔAUC +0.159 (0.061 to 0.262)
M2—seniority and phenotype	−83.6	175.2/187.5	0.640 (0.538–0.742)	0.606	Phenotype vs. M0: LR = 3.6 (2; 0.165); ΔAUC +0.042 (−0.025 to 0.115)
M3—seniority, role and phenotype	−70.5	153.0/171.5	0.767 (0.663–0.871)	0.733	Phenotype vs. M1: LR = 2.8 (2; 0.248); ΔAUC +0.010 (−0.021 to 0.047). Role vs. M2: LR = 26.2 (2; <0.001); ΔAUC +0.127 (0.041 to 0.221)
**Perceived educational benefit (secondary)**					
M0—seniority only	−79.2	162.4/168.6	0.571 (0.460–0.682)	0.548	reference
M1—seniority and role	−65.7	139.4/151.7	0.750 (0.645–0.855)	0.722	Role vs. M0: LR = 27.0 (2; <0.001); ΔAUC +0.179 (0.067 to 0.291)
M2—seniority and phenotype	−73.6	155.2/167.5	0.684 (0.585–0.783)	0.657	Phenotype vs. M0: LR = 11.2 (2; 0.004); ΔAUC +0.113 (0.019 to 0.211)
M3—seniority, role and phenotype	−63.4	138.8/157.3	0.818 (0.737–0.898)	0.777	Phenotype vs. M1: LR = 4.5 (2; 0.103); ΔAUC +0.068 (−0.006 to 0.146). Role vs. M2: LR = 20.4 (2; <0.001); ΔAUC +0.134 (0.038 to 0.236)
**Perceived patient-safety benefit (secondary)**					
M0—seniority only	−65.0	134.0/140.2	0.601 (0.474–0.728)	0.566	reference
M1—seniority and role	−46.6	101.2/113.5	0.855 (0.780–0.929)	0.829	Role vs. M0: LR = 36.8 (2; <0.001); ΔAUC +0.254 (0.132 to 0.376)
M2—seniority and phenotype	−61.6	131.2/143.5	0.686 (0.575–0.797)	0.652	Phenotype vs. M0: LR = 6.8 (2; 0.033); ΔAUC +0.085 (−0.009 to 0.187)
M3—seniority, role and phenotype	−46.0	104.0/122.5	0.866 (0.789–0.943)	0.828	Phenotype vs. M1: LR = 1.2 (2; 0.541); ΔAUC +0.011 (−0.014 to 0.045). Role vs. M2: LR = 31.2 (2; <0.001); ΔAUC +0.180 (0.079 to 0.290)

All models were fitted in the full sample of 161 respondents, and every model contains a seniority band, so that the increments for role (2 df: resident and specialist indicators, nurse as reference) and for phenotype (2 df: class 2 and class 3 indicators, class 1 as reference) each isolate a single construct. Likelihood-ratio tests are the primary basis for assessing incremental contribution; the four primary tests (the phenotype and role increments for the two composite outcomes) had Holm-adjusted *p*-values of 0.496, <0.001, 0.496 and <0.001, respectively. Apparent C-statistics are in-sample; optimism-corrected values are from Harrell’s bootstrap procedure with 200 resamples [39]. ΔAUC is the difference in apparent C-statistic, with a 95% percentile confidence interval from 1000 bootstrap resamples in which both models were re-fitted; DeLong tests are not used because they are invalid for nested models evaluated in the same sample [38]. The composite-outcome models were primary and item-specific models secondary; Benjamini–Hochberg q-values for the two secondary phenotype increments after seniority and role (educational and safety benefit) were 0.206 and 0.541. Abbreviations: AIC, Akaike information criterion; AUC, area under the receiver operating characteristic curve; BIC, Bayesian information criterion; CI, confidence interval; DBI, Digital Benefit Index; df, degrees of freedom; LR, likelihood ratio; M0–M3, models as defined in Section 2.4; UI, Uncertainty Index.

For broad perceived benefit, adding phenotype to the seniority-only model yielded a likelihood-ratio statistic of 3.8 (2 df; *p* = 0.150), and adding it to the seniority-and-role model yielded 2.4 (2 df; *p* = 0.301; Holm-adjusted *p* = 0.496), with a ΔAUC of 0.010 whose bootstrap confidence interval (−0.019 to 0.044) included zero. Adding role to the seniority-only model yielded 25.0 (2 df; *p* < 0.001) and adding it to the seniority-and-phenotype model yielded 23.6 (2 df; *p* < 0.001), with a ΔAUC of 0.138 (0.052 to 0.229). AIC and BIC were higher for the combined model than for the seniority-and-role model because the likelihood gain was insufficient to offset the penalty for two additional parameters. The same asymmetry was observed for high uncertainty (phenotype increments of 3.6 and 2.8; role increments of 27.0 and 26.2) and for patient-safety benefit (6.8 and 1.2 versus 36.8 and 31.2). For educational benefit, phenotype improved the seniority-only model (11.2; *p* = 0.004) but not the seniority-and-role model (4.5; *p* = 0.103; q = 0.206); the ΔAUC of 0.068 for that increment had a bootstrap 95% confidence interval of −0.006 to 0.146 and shrank to 0.055 after optimism correction, so it is not interpreted as evidence of an incremental phenotype contribution. Optimism-corrected C-statistics were 0.02–0.04 lower than apparent values and preserved the ordering of the models. BIC selected the seniority-and-role model over the combined model for all four outcomes.

**Table 8:** Adjusted odds ratios (95% CI) for the primary outcomes from the seniority-and-role model (M1) and the combined model (M3).

Predictor	Broad Perceived Benefit, M1	Broad Perceived Benefit, M3	High Uncertainty, M1	High Uncertainty, M3
Resident vs. nurse	7.6 (3.0–19.2); *p* < 0.001	6.9 (2.6–18.3); *p* < 0.001	0.13 (0.05–0.33); *p* < 0.001	0.14 (0.05–0.37); *p* < 0.001
Specialist vs. nurse	5.9 (2.3–15.0); *p* < 0.001	5.1 (1.9–13.7); *p* = 0.001	0.17 (0.07–0.44); *p* < 0.001	0.19 (0.07–0.52); *p* = 0.001
Seniority band, per level	0.84 (0.59–1.20); *p* = 0.34	0.86 (0.60–1.24); *p* = 0.42	1.21 (0.85–1.72); *p* = 0.29	1.18 (0.82–1.70); *p* = 0.38
Class 2 vs. class 1	—	0.72 (0.28–1.86); *p* = 0.50	—	1.35 (0.52–3.51); *p* = 0.54
Class 3 vs. class 1	—	0.48 (0.18–1.27); *p* = 0.14	—	2.02 (0.76–5.36); *p* = 0.16

Values are odds ratios (95% Wald confidence interval) and *p*-values from ordinary maximum-likelihood logistic regression; nurse and class 1 (comprehensive adopters) are the reference categories, and seniority band is entered as an ordinal score (0–5, 6–10, 11–20, >20 years). The two class coefficients are the parameters tested jointly by the 2-df phenotype increment in Table 7. Abbreviations: CI, confidence interval; M1, M3, models as defined in Section 2.4.

In the combined models (Table 8), residents and specialists had five-to-seven-fold higher odds of broad perceived benefit than nurses and 81–86% lower odds of high uncertainty, with confidence intervals excluding 1 in every case, whereas the two class coefficients were imprecise and included 1 (odds ratios of 0.48–0.72 for benefit and 1.35–2.02 for uncertainty). Adding phenotype changed the role coefficients by less than 0.15 on the log-odds scale, consistent with the small likelihood-ratio increments.

### 3.8. Sensitivity of the Role-Versus-Phenotype Comparison to Class Enumeration, Classification Error and Indicator Set (H2 and H3)

Table 9 repeats the two primary increments of Table 7 under alternative class solutions, under classification-error-corrected (BCH) and posterior-probability-weighted estimation, and with a use-only latent class model. The two-class solution contained 101 and 60 respondents; the four-class solution contained 61, 44, 42 and 14 respondents and split class 1 into two profiles differing mainly in the breadth of educational-resource use. The use-only model, fitted to the seven reported-use indicators, retained three classes for comparability (entropy of 0.781; 88, 41 and 32 respondents); its modal classification agreed moderately with the primary classification (Cohen’s kappa = 0.58).

**Table 9:** Sensitivity of the primary role-versus-phenotype increments to class enumeration, classification-error correction and indicator set.

Specification	Broad Perceived Benefit: Phenotype After Seniority and Role, LR (df; *p*)	Broad Perceived Benefit: Role After Seniority and Phenotype, LR (df; *p*)	High Uncertainty: Phenotype After Seniority and Role, LR (df; *p*)	High Uncertainty: Role After Seniority and Phenotype, LR (df; *p*)
Three classes, modal assignment (primary analysis)	2.4 (2; 0.301)	23.6 (2; <0.001)	2.8 (2; 0.248)	26.2 (2; <0.001)
Three classes, BCH three-step	2.9 (2; 0.235)	22.1 (2; <0.001)	3.3 (2; 0.192)	24.7 (2; <0.001)
Three classes, posterior-probability weighting	2.6 (2; 0.273)	23.0 (2; <0.001)	3.0 (2; 0.223)	25.4 (2; <0.001)
Two classes, modal assignment	1.1 (1; 0.294)	24.9 (2; <0.001)	1.6 (1; 0.206)	27.5 (2; <0.001)
Two classes, BCH three-step	1.4 (1; 0.237)	23.8 (2; <0.001)	1.9 (1; 0.168)	26.1 (2; <0.001)
Four classes, modal assignment	4.1 (3; 0.251)	22.4 (2; <0.001)	4.6 (3; 0.204)	25.0 (2; <0.001)
Four classes, BCH three-step	4.7 (3; 0.195)	21.3 (2; <0.001)	5.2 (3; 0.158)	23.6 (2; <0.001)
Use-only indicators (7 items), three classes, modal assignment	1.7 (2; 0.427)	24.3 (2; <0.001)	2.1 (2; 0.350)	26.8 (2; <0.001)
Use-only indicators (7 items), three classes, BCH three-step	2.0 (2; 0.368)	23.5 (2; <0.001)	2.4 (2; 0.301)	25.9 (2; <0.001)

Each row re-estimates models M0–M3 of Table 7 under the stated specification; the two increments shown correspond to H2 (phenotype after seniority and role) and H3 (role after seniority and phenotype). LR, likelihood-ratio statistic with degrees of freedom and unadjusted *p*-value; for the primary specification, the Holm-adjusted *p*-values across the four primary tests were 0.496, <0.001, 0.496 and <0.001. BCH, bias-adjusted three-step estimation with classification-error weights and 500 bootstrap resamples [34,35]; posterior-probability weighting, each respondent contributes to every class in proportion to posterior membership probability. The use-only model was fitted to the seven reported-use indicators and excludes the five perceived-efficiency items. Abbreviations: BCH, Bolck–Croon–Hagenaars; df, degrees of freedom; LR, likelihood ratio.

Across all nine specifications, the phenotype increment after seniority and role was small and non-significant (likelihood-ratio 1.1–5.2; *p* ≥ 0.158), whereas the role increment after seniority and phenotype was large and significant throughout (21.3–27.5; all *p* < 0.001). Classification-error correction slightly increased the phenotype increment, as expected when the attenuation produced by modal assignment is removed, but did not change its inferential status, and the use-only model, which contains no perceived-efficiency content, produced the smallest phenotype increments of all. Shapley shares recomputed under the BCH three-class specification allocated 66.1% and 60.4% of the partitioned pseudo-R^2^ to role and 13.5% and 12.9% to phenotype for broad benefit and high uncertainty, respectively, close to the primary values in Table 11. The conclusion that role carries more explanatory information than phenotype therefore did not depend on the number of classes, on the treatment of classification uncertainty or on the inclusion of perceived-efficiency indicators.

### 3.9. The Domain Hierarchy of Perceived Benefit (Exploratory)

**Figure 3:**
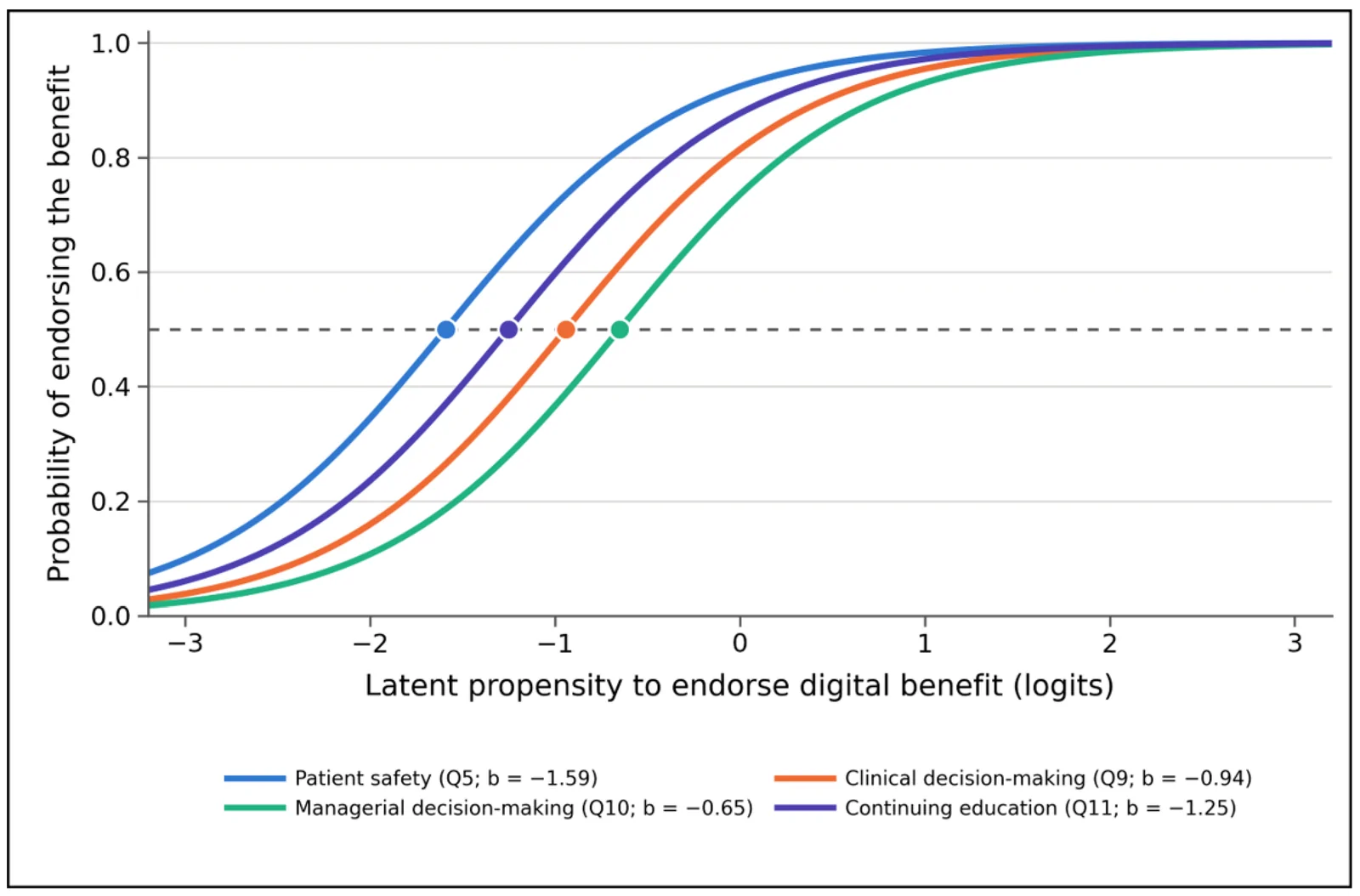
Rasch item characteristic curves for the four benefit domains; markers indicate the difficulty parameter, the point at which endorsement becomes more likely than not; the dashed horizontal line marks the 0.5 probability level.

The exploratory Rasch item hierarchy and descriptive nurse–physician DIF contrasts are reported in Table 10 and Figure 3.

**Table 10:** Exploratory Rasch analysis of the four benefit domains, with descriptive DIF contrasts between nurses and physicians.

Benefit Domain	Endorsed, %	Difficulty (Logits)	95% CI	Infit MSQ	Outfit MSQ	DIF (Logits)
Patient safety (Q5)	85.7	−1.59	−2.05 to −1.13	0.97	0.64	+0.59
Continuing education (Q11)	80.1	−1.25	−1.65 to −0.86	0.85	0.58	+0.43
Clinical decision-making (Q9)	73.9	−0.94	−1.29 to −0.60	0.73	0.52	−0.22
Managerial decision-making (Q10)	67.1	−0.65	−0.97 to −0.33	0.84	0.65	−0.80

Items are ordered from easiest to hardest to endorse. The Rasch model estimated a common discrimination of 1.57 (95% CI 1.06 to 2.09); allowing item-specific discrimination did not improve fit (likelihood-ratio χ^2^ = 5.3, 3 df, *p* = 0.150; AIC 375.0 versus 374.3). Infit and outfit mean-square statistics were within the conventional 0.5–1.5 range. DIF is the centred nurse-minus-physician item-difficulty contrast; a positive value indicates that the item was relatively harder for nurses than their average item. Person separation reliability was 0.07, so individual person measurement is not interpretable. DIF standard errors, confidence intervals, and *p*-values were not available, and no multiplicity adjustment was performed; the DIF values are exploratory descriptive contrasts, not evidence of statistically established group differences. Abbreviations: CI, confidence interval; DIF, differential item functioning; MSQ, mean square.

In the fitted four-item Rasch model, patient safety had the lowest estimated difficulty (−1.59 logits), followed by continuing education (−1.25), clinical decision-making (−0.94), and managerial decision-making (−0.65). The item-level fit statistics did not indicate gross misfit, but the person separation reliability of 0.07 means that the scale cannot distinguish respondents reliably. The centred DIF contrasts were +0.59 logits for patient safety, +0.43 for continuing education, −0.22 for clinical decision-making, and −0.80 for managerial decision-making. Without uncertainty intervals or multiplicity-adjusted tests, these values suggest possible domains for future study but cannot establish differential functioning between nurses and physicians.

Figure 3 displays the fitted item characteristic curves and makes the practical meaning of the difficulty parameters legible. Because the Rasch model constrains all four curves to a common slope, they are parallel and differ only in horizontal position, and the markers at the 0.5 probability level identify the latent propensity at which each domain becomes more likely than not to be endorsed. The span between the easiest and hardest domain is 0.94 logits, which at the estimated discrimination of 1.57 corresponds to a substantial difference in endorsement probability: a respondent sitting at the mean of the latent scale has a fitted probability of 0.92 of endorsing the patient-safety benefit but only 0.74 of endorsing the managerial benefit.

### 3.10. Exploratory Decomposition of the Nurse–Physician Contrast and Partitioning of Model Information (H4)

The exploratory descriptive decomposition (H4) and the secondary Shapley partitioning are reported in Table 11.

**Table 11:** Exploratory descriptive decomposition of the nurse–physician contrast and hierarchical partitioning of McFadden’s pseudo-R^2^.

Parameter	Estimate	95% CI	*p*-Value
**Panel A. Exploratory descriptive decomposition of the nurse–physician contrast (risk difference, percentage points)**			
Broad perceived benefit—total contrast (model-based)	36.3	19.1 to 50.9	<0.003
Broad perceived benefit—direct component	35.0	17.0 to 51.5	<0.003
Broad perceived benefit—indirect component via technology-adoption score	1.2	−3.4 to 7.7	0.598
Broad perceived benefit—indirect share of total contrast via adoption, % (descriptive)	3.4	−10.3 to 23.8	0.598
Broad perceived benefit—indirect component via class 1 (comprehensive-adopter) probability	−1.5	−7.6 to 6.4	0.662
Broad perceived benefit—indirect share of total contrast via phenotype, % (descriptive)	−4.1	−23.4 to 21.1	0.662
High uncertainty—total contrast (model-based)	−33.9	−48.1 to −19.4	<0.003
High uncertainty—direct component	−32.7	−48.1 to −17.1	<0.003
High uncertainty—indirect component via technology-adoption score	−1.2	−6.5 to 3.1	0.615
High uncertainty—indirect share of total contrast via adoption, % (descriptive)	3.5	−9.3 to 21.7	0.615
**Panel B. Hierarchical partitioning: Shapley allocation of total McFadden pseudo-R^2^**			
Broad perceived benefit—professional role	0.121	68.3% of partitioned total	—
Broad perceived benefit—digital-use/efficiency phenotype	0.021	11.9% of partitioned total	—
Broad perceived benefit—technology-adoption score	0.018	10.2% of partitioned total	—
Broad perceived benefit—seniority	0.017	9.7% of partitioned total	—
High uncertainty—professional role	0.128	62.3% of partitioned total	—
High uncertainty—digital-use/efficiency phenotype	0.024	11.4% of partitioned total	—
High uncertainty—technology-adoption score	0.022	10.6% of partitioned total	—
High uncertainty—seniority	0.032	15.7% of partitioned total	—

Panel A reports an exploratory model-based decomposition on the risk-difference scale for the nurse–physician contrast, with residents and specialists combined as physicians and adjustment for seniority band; 800 bootstrap replications with 400 intermediate-variable simulations each were used. The direct and indirect components are descriptive partitions of an observed, model-based association; they are not natural direct or indirect effects and carry no causal interpretation in this cross-sectional design. With 800 bootstrap replications, the smallest attainable two-sided *p*-value is 0.0025, reported as <0.003. Benjamini–Hochberg q-values for the four indirect components were all >0.60. Panel B reports each predictor block’s Shapley allocation of total McFadden pseudo-R^2^, averaged across all entry orders. The percentages are shares of the partitioned pseudo-R^2^, not percentages of outcome variance or causal importance. Phenotype and adoption are correlated constructs, so block allocations are not independent mechanisms. Abbreviations: CI, confidence interval.

For broad perceived benefit, the model-based nurse–physician total contrast was 36.3 percentage points (95% CI 19.1 to 50.9). The indirect component through technology adoption was 1.2 points (95% CI −3.4 to 7.7), and the indirect component through class 1 (comprehensive-adopter) probability was −1.5 points (95% CI −7.6 to 6.4). For high uncertainty, the total contrast was −33.9 points (95% CI −48.1 to −19.4), while the indirect component via adoption was −1.2 points (95% CI −6.5 to 3.1). Thus, the exploratory decompositions provided little evidence that the nurse–physician contrast was carried by adoption breadth or by phenotype probability; they describe an observed association and do not identify a causal direct role effect. In the Shapley partitioning, professional role received 0.121 of the total McFadden pseudo-R^2^ = 0.178 for perceived benefit (68.3% of the partitioned total), compared with 11.9% for phenotype, 10.2% for adoption and 9.7% for seniority. For high uncertainty, role received 62.3% of the partitioned total.

## 4. Discussion

### 4.1. Analysis of Findings

The four hypotheses are considered in turn before the broader interpretation.

H1, that the 12 indicators would yield at least two interpretable phenotypes crossing professional boundaries, was supported. In the selected three-class representation, 16% of nurses fell in class 1 (comprehensive adopters) and 30% of physicians fell outside the class that dominated their profession, and class 3 drew members from all three groups in appreciable numbers; the two- and four-class solutions likewise placed a minority of each profession with the majority of the other. This pattern is what the diffusion-of-innovations account predicts, namely adopter categories defined by behaviour that cut across occupational titles [7], and it agrees with intensive care studies reporting wide variation in usability ratings and reported problems within, not only between, nursing and medical staff [25,26]. The phenotypes are nonetheless an exploratory description: the enumeration criteria disagreed, class 3 had the lowest mean posterior probability (0.83), and the profiles combine reported use with perceived efficiency, so they should be treated as a working typology to be tested in other departments rather than as an established classification.

H2, that phenotype would add information beyond seniority and role, was not supported for the primary outcomes, and the sensitivity analyses in Table 9 show that this was not a consequence of the class-enumeration decision, of classification error in modal assignment, or of the inclusion of perceived-efficiency indicators. Three explanations, each grounded in prior research, appear plausible. First, most of the information carried by phenotype is shared with role: the association between the two was moderate-to-strong (Cramér’s V = 0.439), the seniority-and-phenotype models discriminated above chance (C-statistics 0.63–0.69), and phenotype improved seniority-only models for two of the four outcomes, so phenotype is not uninformative but largely redundant once role is known. This is consistent with implementation reviews that locate the determinants of adoption in role-specific work arrangements [8,45,46,47] and with the FITT framework, in which the fit between task and technology, not the breadth of use, governs perceived benefit [27]. Second, the technology-acceptance literature has repeatedly shown that perceived usefulness depends on whether a system supports the user’s own tasks rather than on how many systems are used [2,3,48,49]; in intensive care specifically, information systems save documentation time for nurses but yield inconsistent gains for physicians [21,22], and usability problems concentrate in medication presentation and documentation steps that fall differently on the two groups [26]. A profile that counts tools and perceived efficiencies across the department therefore need not track the benefit an individual experiences in their own tasks. Third, statistical power was limited: with 161 respondents, the 2-df likelihood-ratio test had roughly 80% power to detect an increment of about 0.05 in McFadden pseudo-R^2^, whereas the observed phenotype increment was about 0.01, so a small genuine contribution cannot be excluded. What can be said is that any such contribution is much smaller than that of role, and that the direction of the construct overlap between the efficiency indicators and appraisal would, if anything, have inflated rather than masked it.

H3, that role would add information beyond phenotype, was supported: adding role to seniority-and-phenotype models produced large improvements for every outcome (likelihood-ratio of 20.4–31.2), and the ranking of the two constructs was unchanged under every sensitivity specification and in the Shapley partitioning. These comparisons indicate where explanatory information was concentrated in this sample; they do not show that professional role itself caused appraisal differences. A role indicator may proxy task allocation, system access, type of exposure and visibility of digital outputs within the local work system [50]. Reviews of user-group barriers and organisational implementation likewise show that technology is encountered through role-specific work arrangements rather than as a uniform exposure [45,46,47]. Syntheses covering the post-pandemic period reach the same conclusion: the barriers and facilitators that govern professionals’ use of digital tools cluster around workflow fit, training and organisational support, all of which are distributed unevenly across roles [4], and human-factors guidance for clinical AI stresses that a tool’s value is realised only through role-specific work design [51].

H4, that adoption breadth or class 1 probability would show an indirect association linking physician status to appraisal, was not supported: the indirect components were within two percentage points of zero with confidence intervals spanning zero, and the direct component was almost identical to the total contrast. This is the decompositional counterpart of the H2 result. It is consistent with self-efficacy and acceptance research showing that availability and use do not translate directly into perceived usefulness [48,52,53], and with evidence that nurses exposed to the same records as physicians report more stress and less benefit when training and workflow fit are insufficient [12,13]. The decomposition is descriptive, however: with cross-sectional data and self-reported use, it cannot distinguish an absent pathway from one that operates in both directions.

The findings provide little evidence that broader reported digital engagement was sufficient to close appraisal differences, but this should not be read as evidence that exposure is irrelevant. Professional role may capture which system component is used, whether use is discretionary or mandatory, the burden attached to it, and whether downstream benefits are visible. Self-efficacy and technology-acceptance research also suggests that availability and use do not fully determine perceived usefulness [52,53]. The exploratory Rasch pattern raises the possibility that benefit domains differ in visibility across groups, but the four-item scale had very low person reliability, and the DIF contrasts lacked inferential uncertainty. Accordingly, access to interpretation is one plausible explanation among several, not a demonstrated mechanism.

Any implementation implication should be treated as a hypothesis for prospective evaluation. Widening access and training may remain necessary, but role-specific interpretive support, feedback linking digital activity to patient or operational outcomes, and redesign of burdensome workflows could be tested as complementary strategies rather than presented as demonstrated recommendations. Reviews report that electronic records can disrupt nurses’ cognitive work and have mixed effects on nursing time [54,55]; more recent work quantifies this burden, with documentation burden substantial for physicians and nurses alike but measured differently in each group [11], burnout attributed to electronic record use in roughly 40% of health professionals in pooled estimates [56], and record-related stress among nurses linked to insufficient training and workflow disruption [12,57], while acceptance research emphasises perceived usefulness and self-efficacy [48,49]. Nursing informatics scholarship argues for stronger nursing participation in data interpretation and design [58], and critical-care cognitive task analysis supports attention to role-specific information needs [59]. Studies of digital workload and health-IT-related stress further caution that more exposure can also mean more burden [60,61]. In anaesthesia and intensive care specifically, physicians surveyed before and during the introduction of AI-based decision support express cautious optimism that is conditional on transparency, external validation and integration into existing workflows [18,19,20], and implementation reviews of critical-care AI identify clinician acceptance as a prerequisite for any benefit to be realised [15,16]. These observations are consistent with the present finding that role, as a proxy for task context, carried more information about appraisal than reported use did. These sources support the plausibility of the proposed tests, not their effectiveness in this sample; implementation should remain sociotechnical and context-sensitive [62].

### 4.2. Study Limitations

Several limitations temper interpretation. First, the cross-sectional design cannot establish temporal order or causal effects; the decomposition analysis is an exploratory descriptive partition of an observed association, not a mediation analysis with causal estimands. Second, the data are self-reported, were collected with a locally developed instrument whose test–retest reliability was not assessed, and come from one tertiary emergency hospital, limiting generalisability and correspondence with observed digital behaviour. Third, class enumeration was uncertain: BIC favoured two classes, other criteria extended improvement to four, and the four-class solution contained a 14-person class. The sensitivity analyses in Table 9 show that the primary comparison did not depend on this choice, but the three-class phenotypes themselves remain an exploratory description rather than an established typology. Fourth, modal assignment ignores classification error; the BCH and posterior-probability-weighted re-analyses gave the same conclusions, although the BCH standard errors rely on bootstrap resampling of a two-stage procedure in a modest sample [33,34,35]. Fifth, the class indicators mix reported use with perceived operational efficiency, creating construct overlap with appraisal-related perceptions; the use-only sensitivity analysis showed that this overlap did not drive the comparison, but phenotype, adoption score, and Resource-Efficiency Index are still not conceptually independent. Sixth, role and seniority are related, and sparse cells limit separate interpretation of their adjusted coefficients. Seventh, discrimination was internally validated by bootstrap only; optimism-corrected C-statistics were 0.02–0.04 lower than apparent values, and external validation in another centre has not been performed. Eighth, the study was not powered to detect small increments: with 161 respondents, the 2-df likelihood-ratio tests had roughly 80% power for an increment of about 0.05 in McFadden pseudo-R^2^ but little power for increments of the size observed for phenotype (about 0.01), so the absence of a phenotype increment should be read as no evidence of a material increment rather than as proof of none. Primary tests were Holm-adjusted and secondary tests false-discovery-rate-adjusted, but the number of exploratory analyses remains large, and those findings are hypothesis-generating. Finally, the four-item Rasch analysis had person separation reliability of 0.07, and DIF contrasts lacked standard errors, confidence intervals, *p*-values, and multiplicity adjustment. The item ordering is descriptive; person estimates and group-specific DIF claims are not reliable. Larger multicentre longitudinal studies should pre-specify class-solution sensitivity analyses and classification-error correction as done here, add external validation, objective usage measures and adequately powered item-level measurement, and measure reported use and perceived efficiency with separate instruments.

## 5. Conclusions

Among 161 anaesthesia and intensive care professionals, the exploratory three-class digital-use/efficiency solution crossed professional boundaries, but its associations with perceived benefit were modest. With seniority held in every model, professional role added substantially more explanatory information than phenotype, whereas phenotype added little after seniority and role for the primary outcomes; this ranking was unchanged under two- and four-class solutions, classification-error correction, use-only indicators and internal validation. The phenotype findings themselves remain exploratory, and the exploratory decompositions provided little evidence that the role contrast was carried by adoption breadth or phenotype probability. These cross-sectional findings do not establish that role causes appraisal differences or that broader engagement cannot matter. They instead motivate prospective evaluation of role-specific exposure type, workflow burden, visibility of system outputs, and interpretive support as possible explanations for appraisal gaps.

## Data Availability

The anonymized minimal respondent-level dataset and the analysis scripts supporting the findings are available from the corresponding author upon reasonable request. Public deposition of the participant-level dataset is restricted because data sharing remains subject to the ethical and institutional requirements governing the study.

## Appendix A

**Survey instrument.** The questionnaire was administered on paper in Romanian in a single wave (4 April to 30 June 2022); the English translation below preserves the item order and response options. Items marked with an asterisk supplied the 12 latent class indicators; Q5, Q9, Q10 and Q11 are the four benefit-appraisal items that were held outside the class-defining model.

**Section A. Respondent characteristics.** Q1. Your professional role in the department: nurse/resident physician/specialist physician. Q2. Years of professional experience in anaesthesia and intensive care: 0–5/6–10/11–20/more than 20. Q3. Do you use digital tools (patient monitoring, electronic records, planning software or similar) in your daily work? yes/no.

**Section B. Technologies used and patient safety.** Q4.* Which of the following digital technologies do you use in your work? (tick all that apply): patient-monitoring systems; electronic health records; anaesthesia-planning software; training simulators. Q5. In your opinion, have digital technologies improved patient safety in your department? yes/no/cannot appraise.

**Section C. Educational resources and barriers.** Q6.* Which of the following digital educational resources have you used in the past 12 months? (tick all that apply): e-learning platforms; simulation-based training; webinars. Q7. Which of the following barriers limit your use of digital technologies? (tick all that apply): lack of training; technical problems; resistance to change.

**Section D. Perceived operational efficiency.** Q8.* Since the introduction of digital technologies, which of the following resources are used more efficiently in your department? (tick all that apply): medications; medical staff; sanitary materials; bed occupancy; protective equipment.

**Section E. Benefit appraisal.** Q9. Have digital technologies improved clinical decision-making in your department? yes/no/cannot appraise. Q10. Have digital technologies improved managerial decision-making in your department? yes/no/cannot appraise. Q11. Have digital technologies improved continuing education in your department? yes/no/cannot appraise.

**Scoring.** Technology-adoption score = number of technologies ticked in Q4 (0–4); Barrier Burden score = number of barriers ticked in Q7 (0–3); Resource-Efficiency Index = number of resources ticked in Q8 (0–5); Digital Benefit Index = number of ‘yes’ responses to Q5, Q9, Q10 and Q11 (0–4); Uncertainty Index = number of ‘cannot appraise’ responses to the same four items (0–4). Broad perceived benefit was defined as a Digital Benefit Index of at least 3 and high uncertainty as an Uncertainty Index of at least 2.
